# Deep learning for Dixon MRI-based attenuation correction in PET/MRI of head and neck cancer patients

**DOI:** 10.1186/s40658-022-00449-z

**Published:** 2022-03-16

**Authors:** Anders B. Olin, Adam E. Hansen, Jacob H. Rasmussen, Björn Jakoby, Anne K. Berthelsen, Claes N. Ladefoged, Andreas Kjær, Barbara M. Fischer, Flemming L. Andersen

**Affiliations:** 1 Department of Clinical Physiology, Nuclear Medicine and PET & Cluster for Molecular Imaging, Rigshospitalet, University of Copenhagen, Blegdamsvej 9, 2100 Copenhagen, Denmark; 2 Faculty of Health and Medical Science, University of Copenhagen, Copenhagen, Denmark; 3 Department of Radiology, Rigshospitalet, University of Copenhagen, Copenhagen, Denmark; 4 Department of Otorhinolaryngology, Head and Neck Surgery and Audiology, Rigshospitalet, University of Copenhagen, Copenhagen, Denmark; 5 Siemens Healthcare GmbH, Erlangen, Germany; 6 University of Surrey, Guildford, Surrey UK; 7 Department of Oncology, Section of Radiotherapy, Rigshospitalet, University of Copenhagen, Copenhagen, Denmark; 8 King’s College London and Guy’s and St Thomas’ PET Centre, School of Biomedical Engineering and Imaging Sciences, King’s College London, King’s Health Partners, London, UK

**Keywords:** PET/MRI, MR-AC, Deep learning, Head and neck cancer

## Abstract

**Background:**

Quantitative whole-body PET/MRI relies on accurate patient-specific MRI-based attenuation correction (AC) of PET, which is a non-trivial challenge, especially for the anatomically complex head and neck region. We used a deep learning model developed for dose planning in radiation oncology to derive MRI-based attenuation maps of head and neck cancer patients and evaluated its performance on PET AC.

**Methods:**

Eleven head and neck cancer patients, referred for radiotherapy, underwent CT followed by PET/MRI with acquisition of Dixon MRI. Both scans were performed in radiotherapy position. PET AC was performed with three different patient-specific attenuation maps derived from: (1) Dixon MRI using a deep learning network (PET_Deep_). (2) Dixon MRI using the vendor-provided atlas-based method (PET_Atlas_). (3) CT, serving as reference (PET_CT_). We analyzed the effect of the MRI-based AC methods on PET quantification by assessing the average voxelwise error within the entire body, and the error as a function of distance to bone/air. The error in mean uptake within anatomical regions of interest and the tumor was also assessed.

**Results:**

The average (± standard deviation) PET voxel error was 0.0 ± 11.4% for PET_Deep_ and −1.3 ± 21.8% for PET_Atlas_. The error in mean PET uptake in bone/air was much lower for PET_Deep_ (−4%/12%) than for PET_Atlas_ (−15%/84%) and PET_Deep_ also demonstrated a more rapidly decreasing error with distance to bone/air affecting only the immediate surroundings (less than 1 cm). The regions with the largest error in mean uptake were those containing bone (mandible) and air (larynx) for both methods, and the error in tumor mean uptake was −0.6 ± 2.0% for PET_Deep_ and −3.5 ± 4.6% for PET_Atlas_.

**Conclusion:**

The deep learning network for deriving MRI-based attenuation maps of head and neck cancer patients demonstrated accurate AC and exceeded the performance of the vendor-provided atlas-based method both overall, on a lesion-level, and in vicinity of challenging regions such as bone and air.

## Background

Simultaneous PET/MRI offers great opportunities for cancer research and new possibilities for radiotherapy planning as it provides high anatomical sensitivity from MRI and functional information from both MRI and PET [1, 2]. However, accurate attenuation correction (AC) of PET continues to be a major challenge for PET/MRI and is critical for quantitative analysis but also qualitatively as it affects the visual representation of the PET tracer distribution. Traditionally, PET AC is handled by an attenuation map derived from either a transmission scan using an external rotating rod-source or a CT scan (for PET/CT scanners) converted from Hounsfield units (HU) into linear attenuation coefficients (LAC) at 511 keV. These methods are not accessible for PET/MRI, which instead infers an attenuation map (synthetic CT) from the patient MRI to perform so-called MRI-based AC (MR-AC). MR-AC is inherently challenging because MRI is not linked to photon attenuation information, and it is further complicated by the fact that while air and bone both lack signal in traditional MRI, they have completely different attenuation properties.

Initial commercially available MR-AC methods have traditionally relied on the quickly acquired Dixon MRI sequence, which can provide images for segmentation into air, lung, fat, and soft tissue, each with a predefined LAC value [3]. But attenuation coefficients of bones are not obtained and instead replaced by soft tissue, which has been shown to substantially underestimate PET values by more than 20% in regions closest to bone [4, 5].

Consequently, the strive for improving PET quantification has led to the development of many bone-including MR-AC methods primarily for brain imaging [6–11]. Especially segmentation-based methods using specialized MRI sequences (ultra short echo time (UTE) or zero echo time (ZTE)) obtaining some signal from bone, and atlas-based strategies for which the patient MRI is matched to a CT-atlas have demonstrated excellent results and solved the MR-AC issue for the adult head with normal anatomy [12]. However, the translation of these methods to other body regions is challenged by several factors. First, the scanned object is larger and more prone to motion (e.g., swallowing, respiration and pulsation), which challenges current UTE/ZTE MRI sequences with a small field-of-view and long acquisition times. Second, body regions outside the brain exhibit large inter-patient variations and many non-rigid structures, which are especially problematic for atlas-based methods as the registration requires a high degree of deformability. Third, some regions (e.g., head and neck) have a particular complex anatomy with many different bony structures and air cavities in close vicinity of each other. For such complex areas, atlas-based methods may struggle to find a suitable match and UTE/ZTE segmentation-based are most likely influenced by susceptibility effects, as it has been seen for the sinuses and skull base in brain imaging [13].

Recently, deep learning has emerged as an alternative MR-AC strategy [14] for the brain [15–17] and the pelvic region [18, 19] and has shown accurate and robust performances. Although several studies using deep convolutional neural networks for converting MRI to CT also exist for the head/neck region and have been applied for use in radiotherapy [20–24], only limited effort has been put into detailed evaluation of the effect on PET AC where the lower photon energy (511 keV compared to MeV typically used radiotherapy) increases the sensitivity to wrong tissue attenuation coefficients. The lack of studies in the field is most likely due to difficulties in obtaining accurately aligned PET/MRI and CT data for method development and evaluation.

In this study, we use a unique dataset where PET/MRI and CT imaging of head and neck cancer patients are acquired in matching position ensured by the radiotherapy immobilization devises. We apply a deep learning model – developed for radiotherapy purposes [23, 24] – to derive patient-specific MRI-based attenuation maps using only the standardized and quick Dixon MRI as input and perform a comprehensive evaluation of its performance on PET AC including the effect of bone structures and air cavities. We use CT-based AC as the reference and compare our results to those of the vendor-provided MRI-based method.

## Materials and methods

### Data acquisition

This study analyzed the MR-AC performance on 11 patients (10 males/1 female; 58 ± 9 years old) referred for radiotherapy of head and neck cancer (6 oropharyngeal/3 hypopharyngeal/2 laryngeal cancers; 7 patients with lymph node involvement), where 10 of these were part of a previous study concerning MRI-based radiotherapy dose calculations [24] and one patient was added since.

Each patient underwent a routine planning [^18^F]FDG-PET/CT (Siemens TruePoint 64; Siemens Healthcare GmbH, Erlangen, Germany) and subsequently a PET/MRI (Siemens Biograph mMR with VE11P software; Siemens Healthcare GmbH, Erlangen, Germany) using the same [^18^F]FDG injection. Both PET/CT and PET/MRI were acquired in radiotherapy treatment position using a flat table overlay (Qfix, Avondale, PA), a chest board (XRT-Series 6000; Candor, Gislev, Denmark) and individualized thermoplastic masks (EasyFrame; Candor) as described in [23]. All patients gave written informed consent, and the local ethics committee approved the study (H-7023133).

Patients fasted for a minimum of 4 h prior to injection of [^18^F]FDG (4 MBq/kg) given approximately 60 min prior to the PET/CT scan and an average of 142 min prior to PET/MRI.

CT was acquired with a 100/120 kVp tube voltage with i.v. contrast and reconstructed using the iterative algorithm for metal artifact reduction (iMAR) on 512 × 512 matrices, a pixel spacing 1.52 × 1.52 mm^2^ and a slice thickness of 2 mm.

PET/MRI scanning of the patients in radiotherapy position prevented the use of the standard head/neck coil for MRI, why an alternative coil setup consisting of two flexible coils in coil-holders hovering above the patient head and another flexible coil over the thorax were used, as described in another study [23]. For all patients, the MRI protocol included acquisition of the standard Dixon MRI sequence (flip angle of 10; repetition time of 3.85 ms; first/second echo time of 1.23/2.46 ms; in matrices of 384 × 312 × 88 with voxel size of 1.3 × 1.3 × 3.0 mm^3^) over one bed position covering the tumor. Simultaneously, PET emission data were acquired for an average of 26 ± 7 min in list mode.

### Attenuation maps

For each patient, two different approaches were used for deriving Dixon MRI-based attenuation maps. First using the vendor-provided atlas-based method, which first segments the Dixon MRI into soft tissue, fat, lung tissue, and air [3] followed by superimposing of major bones (hip, spine and skull) from a bone-atlas [25]. The resulting attenuation map was created in 240 × 157 × 130 matrices with voxel size of 2.1 × 2.6 × 2.1 mm^3^. The second approach employed a deep learning network, presented in our previous work [24]. We did not re-train the model for the purpose of this study and refer to the original publication for details regarding network architecture and parameters [24]. In short, it is a convolutional neural network developed in TensorFlow 2.10 [26], with an architecture inspired by the 3D U-net [27, 28] that takes Dixon MRI in-phase and opposed-phase images as inputs and infers an attenuation map in LAC values. The network relies on transfer learning from a similar network trained with > 800 brain patients [16] and was subsequently fine-tuned on a total of 17 head and neck cancer patients (in a leave-one-out process) of which 10 are included in this study and the rest were excluded due to lack of PET data acquisition. Each patient’s attenuation map was inferred after being left out in the training process [24]. For the eleventh patient, who was recruited and scanned since the training of the network, the attenuation map was inferred using a (leave-one-out) model selected at random. Inference was performed on a standard desktop PC with a Titan V GPU (NVIDIA Corporation, Santa Clara, CA) and took less than 10 s per attenuation map, which subsequently was resampled to match the vendor-provided atlas-based attenuation map.

For each patient, a CT-based reference attenuation map was also derived. Non-patient objects (e.g., scanner bed) were removed from the CT images, before they were converted from HU to LAC using a biliary scaling [29]. The CT-based attenuation maps were registered to their corresponding Dixon MRI by an initial rigid registration (reg_aladin, NiftyReg) followed by a deformable registration (reg_f3d, NiftyReg), and alignments were validated by a visual inspection. Similarly, the CT-based attenuation maps were resampled to match the vendor-provided atlas-based attenuation map.

### PET reconstructions

PET images were reconstructed with 3D-OP-OSEM (ordinary Poisson ordered subsets expectation maximization) (4 iterations, 21 subsets, 3 mm full width at half maximum Gaussian post-filtering) on 344 × 344 matrices with pixel size 2.1 × 2.1mm^2^ and a slice thickness of 2 mm using the E7Tools software (Siemens Healthcare GmbH, Erlangen, Germany). For each patient three PET images were reconstructed using different attenuation maps—the vendor-provided atlas-based attenuation map (PET_Atlas_), the deep learning derived attenuation map (PET_Deep_), and the CT-based attenuation map (PET_CT_). All PET reconstructions used the hardware-specific attenuation correction as described in [23] and voxels were converted to standardized uptake value (SUV) normalized by body weight. Reconstructed PET images were resampled to match the voxel size of the attenuation maps for data analysis purposes.

### Data analyses

The overall effect of the MRI-based attenuation maps on the reconstructed PET images was studied by calculating joint histograms between PET_Atlas_ and PET_CT_ and between PET_Deep_ and PET_CT_ for all voxels within the patient volumes, for which the coefficients of determination (*R*^2^) were assessed. Keeping PET_CT_ as a reference we calculated the voxelwise relative difference to PET_Atlas_ and PET_Deep_, to estimate the error caused by MR-AC. The distributions of errors were represented in histograms and the absolute errors were shown in cumulative histograms from which we assessed the fraction of voxels with errors less than ± 5%, ± 10% and ± 20%.

To examine the MR-AC methods’ ability to infer bone and air compartments we calculated the Dice coefficients for bone (voxels above 0.11 cm^−1^) and air (not including lung tissue) within the body contour of the patient (voxels below 0.007 cm^−1^). Next, to examine the impact on the PET quantification, we calculated the error in mean SUV (SUV_mean_) within bone and air separately and extended the analysis by assessing the error as a function of spatial distance to the particular compartment. To this end, distance-to-bone maps and distance-to-air maps for each patient were created by first identifying CT voxels being bone or air. Then, for each CT voxel, the transaxial Euclidian distance (2D) to the nearest voxel classified as bone or air was calculated. Voxels outside the patient volume, within lungs and closer to air than bone were excluded in the distance-to-bone maps and vice versa for the distance-to-air maps. The voxels in a distance map was then binned into groups with 3 mm interval and the SUV_mean_ error within each group was calculated for which the lower quartile, median, and upper quartile for each across all patients were reported.

To relate potential shortcomings of MR-AC to anatomical regions, the SUV_mean_ errors in the spinal cord, brain stem, parotid glands, submandibular glands, larynx and esophagus were calculated for each patient. The same calculations were performed for tumors. All regions of interest were delineated for radiotherapy purposes using the images of the PET/CT examination and projected into PET/MRI space using the same transformation as used for the CT (described above). Delineation of all anatomical regions followed the national DAHANCA guidelines [30] and the tumors (primary tumor and involved lymph nodes) were delineated on the PET/CT by an nuclear medicine specialist using Mirada XD software (Mirada Medical, Oxford, United Kingdom) and a visual adaptation of an initial isocontouring starting at 40% of maximum uptake.

## Results

**Fig. 1:**
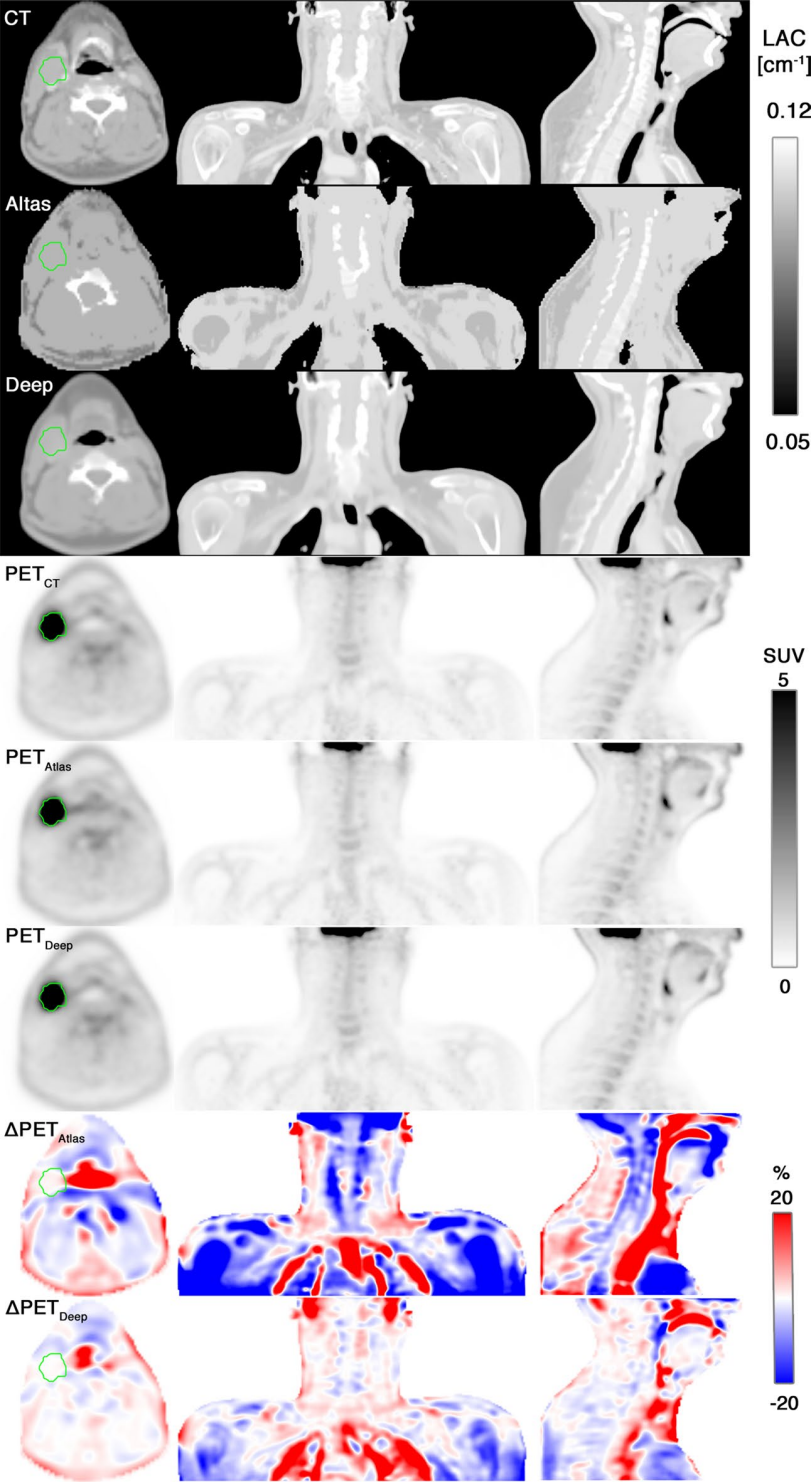
The eleventh patient of the cohort, a 52-year-old male with right base of the tongue cancer and lymph node involvement (T2N1M0). Each row from top to bottom shows an axial, coronal and sagittal slice of: the reference CT; the vendor-provided atlas-based attenuation map (Atlas); the deep learning derived attenuation map (Deep); PET_CT_; PET_Atlas_; PET_Deep_; the relative difference map between PET_CT_ and PET_Atlas_ (ΔPET_Atlas_); the relative difference map between PET_CT_ and PET_Deep_ (ΔPET_Deep_);. The involved lymph node is delineated in green for the axial images. Notice, that the atlas-based attenuation map does not classify the trachea as air and the overall reduced PET error for the deep learning method, which is apparent from the difference maps

Figure 1 shows the attenuation maps, the reconstructed PET images, and the PET voxelwise relative difference maps for patient 11.

**Fig. 2:**
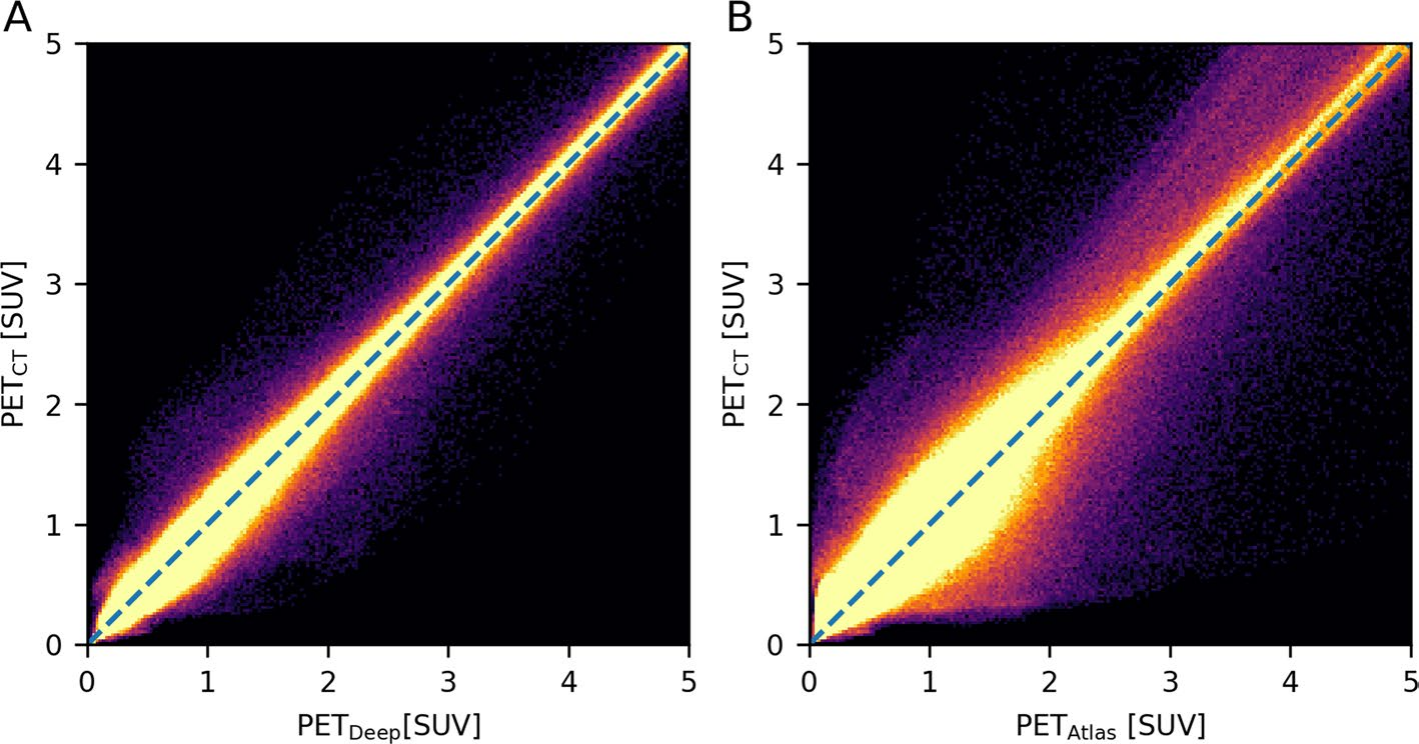
Joint histograms of PET voxels within the patient volumes for (**A**) PET_Deep_ and PET_CT_ (*R*^2^ = 0.997), and (**B**) PET_Atlas_ and PET_CT_ (*R*^2^ = 0.975). Notice, that the axes are clamped to SUV of 5 even though there are higher values in the PET images

**Fig. 3:**
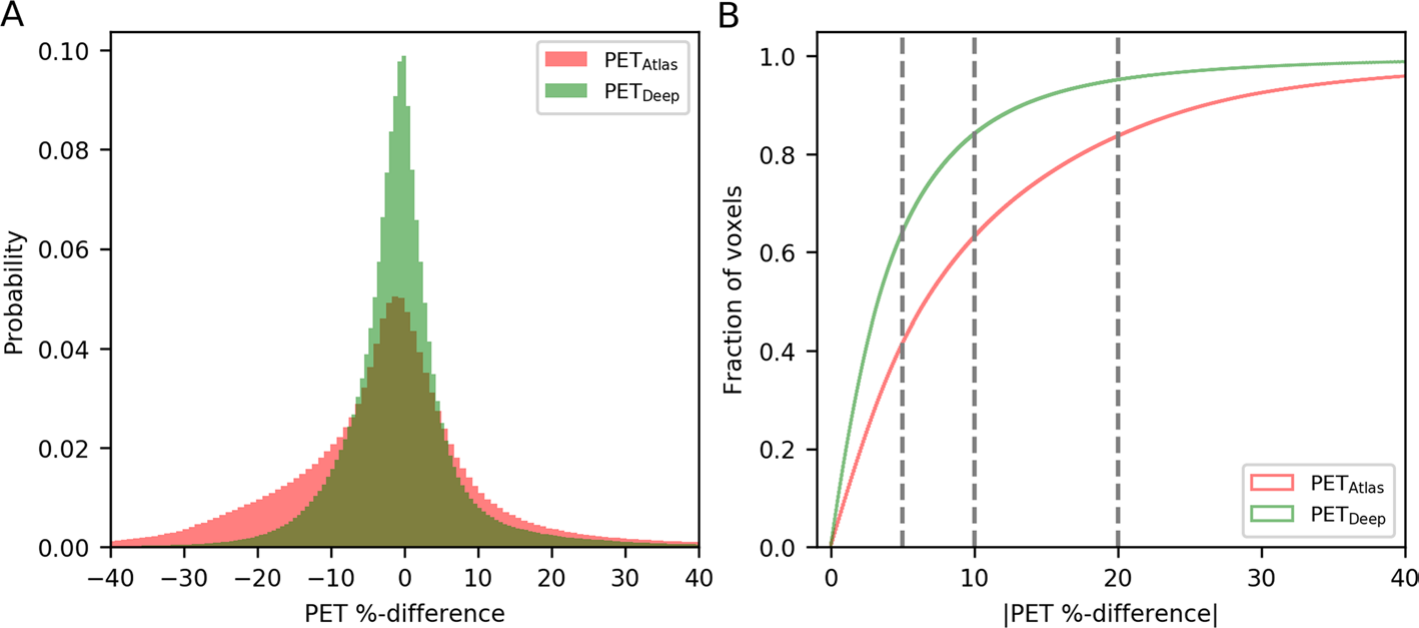
(**A**) Histograms of the PET error distributions for PET_Atlas_ and PET_Deep_. (**B**) Cumulative histogram of the absolute PET error for PET_Atlas_ and PET_Deep_. The vertical dashed lines are located at 5%, 10%, and 20% and the corresponding amount of voxels with errors below these thresholds are 65%, 84%, and 95% for PET_Deep_ and 42%, 64%, and 84% for PET_Atlas_

The joint histograms are shown in Fig. 2, for which the values of PET_Deep_ and PET_CT_ (*R*^2^ = 0.997) are distributed closer to the identity line, than PET_Atlas_ and PET_CT_ (*R*^2^ = 0.975). This can also be seen from the distributions of PET errors within patient volumes as shown in Fig. 3A. For these distributions the mean (± standard deviation) errors are 0.0 ± 11.4% for PET_Deep_ compared to -1.3 ± 21.8% for PET_Atlas_. The cumulative distributions are shown in Fig. 3B, where the fraction of voxels below the thresholds ± 20%, ± 10%, and ± 5% are 95%, 84%, and 65% for PET_Deep_ and 84%, 64%, and 42% for PET_Atlas_.

**Fig. 4:**
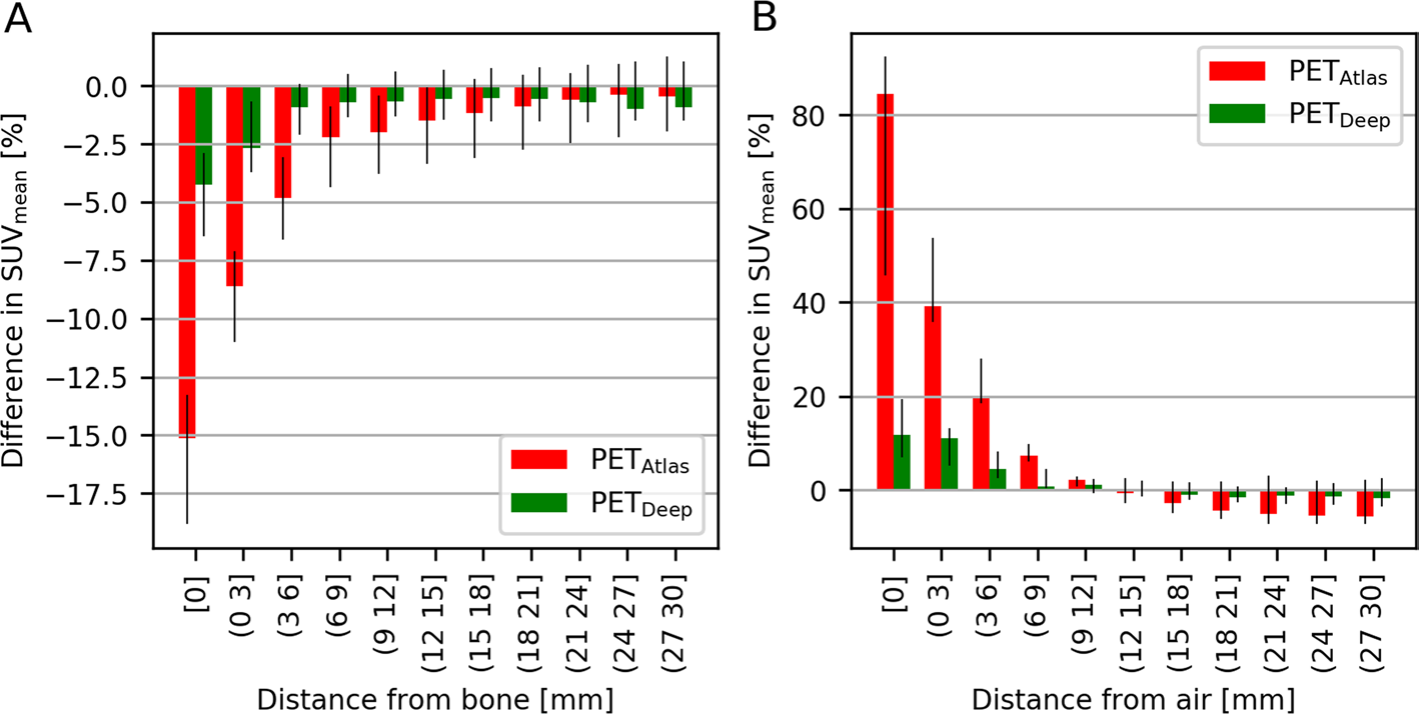
Analysis showing the median error in SUV_mean_ (colored bars) and interquartile range (black errorbars) as a function of distance to either (**A**) bone or (**B**) air

For the atlas-based MR-AC method, the average Dice coefficients for bone and air across all patients are 0.30 ± 0.11 and 0.42 ± 0.16, respectively. Similarly, for the deep learning MR-AC method the Dice coefficients for bone and air are 0.69 ± 0.08 and 0.74 ± 0.08. The PET SUV_mean_ error as a function of distance to bone is shown in Fig. 4A. Within the bone compartment, SUV_mean_ is underestimated by median of − 4% for PET_Deep_ and by -15% for PET_Atlas_. In both cases the PET error decreases with distance to bone, but the interquartile range (error bars) include zero at 3–6 mm from bone for PET_Deep_ and at 18–21 mm from bone for PET_Atlas_. The results of the same analysis for air are seen in Fig. 4B. The median error within air is much lower for PET_Deep_ (12%) than PET_Atlas_ (84%), and the effect decreases with distance similarly to bone.

**Fig. 5:**
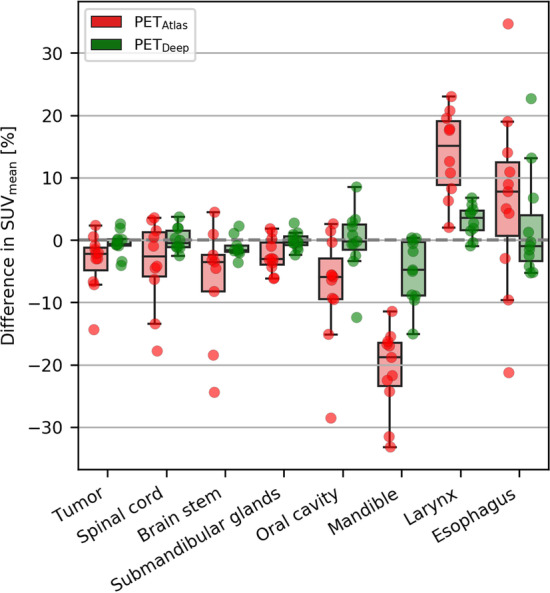
Regional analysis showing box-whiskers plots (box shows the quartiles of the data; whiskers show the 1.5 times interquartile range of the data) of the errors in SUV_mean_ within different anatomical regions and the tumors. The individual errors are shown as colored dots on top of the box-whiskers

The effect of MR-AC on PET quantification in different anatomical regions and within tumors is shown in Fig. 5. The errors for PET_Deep_ are generally lower than for PET_Atlas_ within all of the analyzed regions. For both MR-AC methods the PET uptake in the mandible (jaw bone) is underestimated, whereas the uptake in the larynx (contains air within trachea) is generally overestimated. For PET_Deep_, regions with larger variations are the oral cavity and esophagus. The average (± standard deviation) tumor and involved lymph node volume defined by PET_CT_ is 11.5 ± 10.8 cm^3^ and the differences in SUV_mean_ are − 0.6 ± 2.0% (range: − 4.1%; 2.6%) for PET_Deep_ and − 3.5 ± 4.6% (range: − 14.4%; 2.3%) for PET_Atlas._

**Fig. 6:**
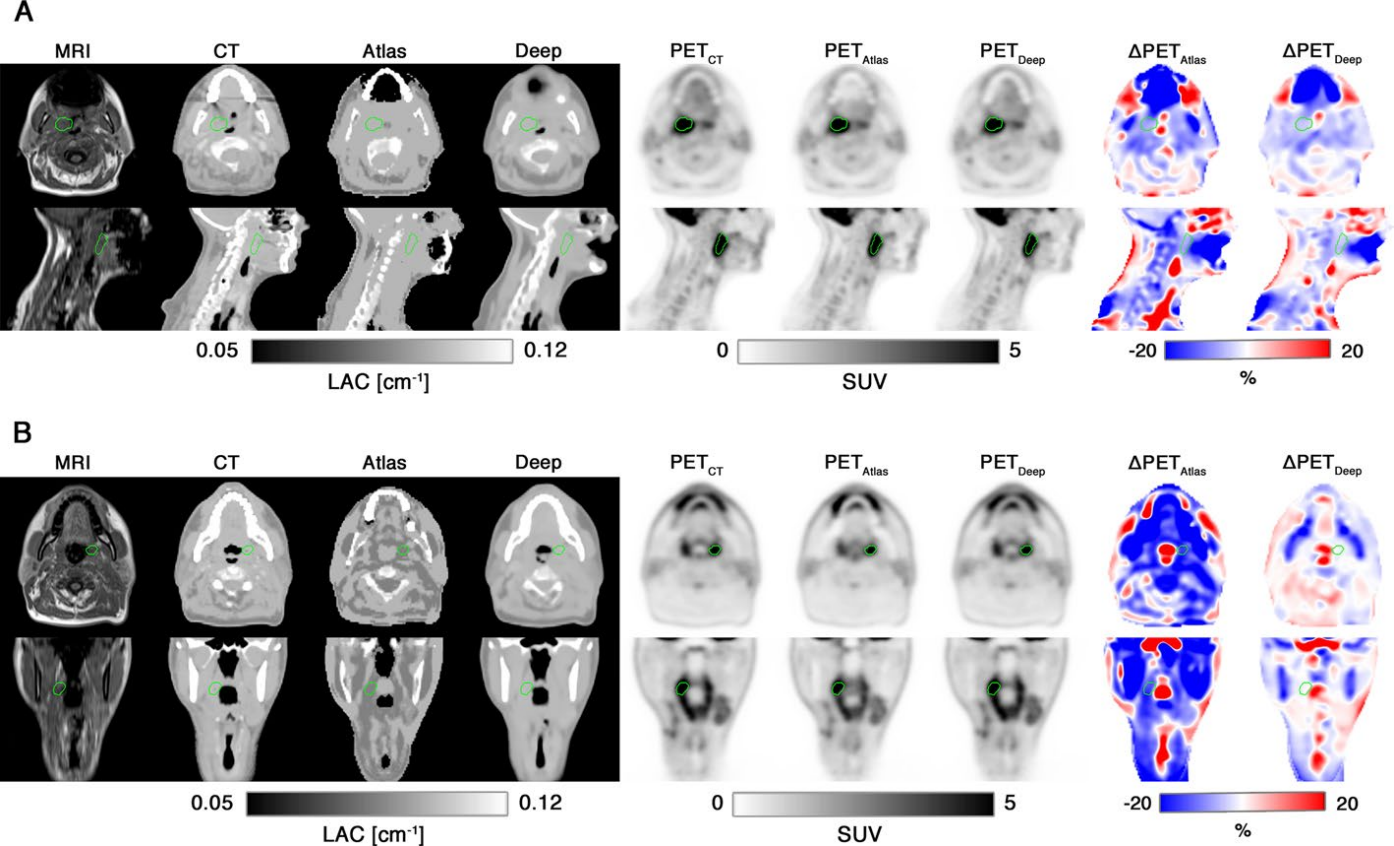
The worst performing cases, based on tumor (green delineation) SUV_mean_ error, for (**A**) the deep learning method (error of −4.1% for PET_Deep_ and −6.7% for PET_Atlas_) and for (**B**) the atlas-based method (error of −0.8% for PET_Deep_ and −14.4% for PET_Atlas_). (**A**) Axial and sagittal slices of a 68 year old female with cancer of the right tonsil and bilateral lymph node involvement (T3N2M0). The large single void in the MRI affects both MR-AC methods. Notice, that the deep learning method partially adds tissue within the MRI signal void, and that the atlas-based method adds the jawbone despite the missing signal. (**B**) Axial and coronal slices 42 year old male with cancer of the left tonsil (T1N1M0). The vendor-provided atlas-based method is affected by a fat–water swap (fat becomes soft tissue and vice versa) and some of the air in trachea is segmented as soft tissue (axial slice)

Figures 6A and B show the two patients with the largest errors in tumor SUV_mean_ for both MR-AC methods.

## Discussion

Accurate MR-AC in the head and neck region is challenging due the complex anatomy with many different bony structures and air cavities often in close vicinity of each other as well as large inter-patient variation. In this study, we evaluated a deep learning network for deriving patient-specific Dixon MRI-based attenuation maps for head and neck cancer patients, which demonstrated small PET errors when using CT-based AC as reference and a performance exceeding the most recent vendor-provided atlas-based MR-AC method.

Quantitatively, our deep learning method provided PET_Deep_ values that were in line with the reference PET_CT_ values and largely corrected for the underestimation that was seen for PET_Atlas_. On a lesion-level, PET_Deep_ showed improved quantification with an average difference (± standard deviation) in SUV_mean_ of − 0.6 ± 2.0% compared to -3.5 ± 4.6% for PET_Atlas_. These findings are in accordance with the results reported in our previous study (SUV_mean_ error of − 0.4 ± 1.2%) [23].

Accurate MR-AC is especially limited by the method’s ability to correctly segment air and bone. The Dice coefficients revealed that the deep learning method exceeded the vendor-provided atlas-based method in this regard. However, because the effects of bone/air on PET quantification are not limited to voxels within bone/air but introduces a spatial varying error, we performed an analysis assessing the PET error as a function of distance to air/bone. We observed that PET errors within the compartments were greatly reduced when using the deep learning method and that for both methods the error rapidly decreases with distance. For PET_Deep_ the bias was only present in the immediate surroundings (3–6 mm), while the bias for PET_Atlas_ had a larger spatial extent (2 cm). These results suggest that the deep learning method will greatly improve the PET accuracy for tumors located in proximity to bone or air and could also have implications for defining the tumor outline. It should be noted that the absolute uptake in the analyzed voxels were mainly very low (especially in air) and that a small absolute difference therefore can appear as a large relative difference.

The results of the regional analysis verified that the largest biases (median errors) were seen for bone (mandible) and regions including air (larynx). When keeping the error-distance relationship in mind it is also worth emphasizing the low errors of PET_Deep_ compared to PET_Atlas_ in the spinal cord, which is surrounded by bone; the oral cavity, where air cavities may be present next to teeth and the jaw; and the esophagus, which is anatomically located posterior to the trachea (air) and anterior to the spine (bone). Although PET_Deep_, in regions like the oral cavity and esophagus showed no clear bias a larger variation was present. Upon visual inspection, this was typically attributed to misclassified air cavities due to e.g., the tongue not being in the same position between the two scans (see sagittal view of the attenuation maps in Fig. 1) and small air cavities in the esophagus not captured on the MRI.

While both MR-AC methods have the strength of relying only on the fast and standardized Dixon MRI sequence, inference of a new patient’s attenuation map by the deep learning approach has the advantage over to the vendor-provided atlas-based method due to several reasons. First, it does not rely on any registration, making the method less sensitive to inter-patient variation and abnormal anatomy. Second, it infers all bones in the body and not just the major bones (hip, spine and skull). Third, the deep learning method proved reliable and robust compared to the vendor-provided method for which we observed frequent artifacts including fat–water swap, incorrect tissue segmentation (especially of air cavities), misplacement of the bone and even failing to add bone (the spine was missing for one patient). While the frequent artifacts are in line with other studies [31–34], the lower MRI quality caused by the altered coil arrangement in this set-up [35] might increase the risk. The deep learning method is, however, more sensitive toward artifacts in the underlying Dixon MR images. In our previous work [24], we reported that approximately half of the patients had metallic dental implants leading to signal voids clearly visible on Dixon MRI and this still holds for this study. The artifacts were generally small (< 2 cm), and the model was to a great extent capable of handling these (see figure in our previous work [24]), but for one patient the model did not fully correct for the presence of a large artifact (Fig. 6A). Improving model robustness toward artifacts could be possible by using a larger and more diverse training cohort [16] and should therefore be of the focus in future work.

Although the deep learning method exceeded the performance of the vendor-provided method, the method still has some remaining PET errors especially surrounding bone, which can most likely be attributed to the underestimation of bone attenuation coefficients/HU (− 199 ± 60 HU) as we reported in our previous study [24]. In a recent deep learning study for AC of the pelvic region, the error in bone was slightly lower (− 1%) compared to ours (− 4%), which could be due to improved prediction of bone attenuation coefficients but a direct comparison should be done with caution due to differences between the two regions. First, the head and neck region (extending from top of skull and down to mid thorax in this study) includes many different and usually smaller bone structures (e.g., skull bones, vertebras of the spine, hyoid bone, shoulder bones and ribs) than seen in pelvic region. Second, unlike the pelvis, the head and neck region is further challenged by the presence of many air/tissue and air/bone interfaces complicating the segmentation task.

Another strategy for AC in PET/MRI, which has become more compelling with the introduction of deep learning, is to ﻿ generate attenuation-corrected PET images directly from uncorrected PET images. Such methods have been applied for whole-body AC with promising results [36, 37].

The primary limitation of this study is that evaluation is only performed on eleven patients and mainly done so by a leave-one-out validation process opposed to a separate test cohort. Furthermore, it would also be desirable to also have a more diverse cohort, e.g., more female patients to assess whether the model performs equally on both genders. Another limiting factor is that the CT served as reference despite not directly reflecting the monoenergetic (511 keV) LAC required for PET and despite being acquired with contrast enhancement, which artificially increases the CT values in some soft tissue regions. Finally, despite the acquisition of both PET/CT and PET/MRI in radiotherapy position using fixation masks, there was still a need for non-rigid registration, for which inaccuracies will affect PET quantification.

## Conclusion

In this study, a deep learning method for deriving patient-specific Dixon MRI-based attenuation maps in the anatomically challenging head and neck region was evaluated for PET AC. Using CT-based AC as reference, the method demonstrated small PET errors in tumors (0.6 ± 2.0%) and a performance exceeding the most recent vendor-provided MR-AC method. The method could have clinical impact, especially on tumor delineations and tumor uptake values close to bone or air compartments.

## Data Availability

The datasets used and/or analyzed during the current study are available from the corresponding author on reasonable request and given approval from relevant regulatory authorities.

## Abbreviations

PET: Positron emission tomography
MRI: Magnetic resonance imaging
CT: Computed tomography
FDG: Fluorodeoxyglucose
OP-OSEM: Ordinary Poisson ordered subsets expectation maximization
AC: Attenuation correction
MR-AC: Magnetic resonance-based attenuation correction
LAC: Linear attenuation coefficient
HU: Hounsfield unit
